# Child sexual offenders show prenatal and epigenetic alterations of the androgen system

**DOI:** 10.1038/s41398-018-0326-0

**Published:** 2019-01-18

**Authors:** Tillmann H. C. Kruger, Christopher Sinke, Jonas Kneer, Gilian Tenbergen, Abdul Qayyum Khan, Alexandra Burkert, Linda Müller-Engling, Harald Engler, Hannah Gerwinn, Nicole von Wurmb-Schwark, Alexander Pohl, Simone Weiß, Till Amelung, Sebastian Mohnke, Claudia Massau, Christian Kärgel, Martin Walter, Kolja Schiltz, Klaus M. Beier, Jorge Ponseti, Boris Schiffer, Henrik Walter, Kirsten Jahn, Helge Frieling

**Affiliations:** 1 Department of Psychiatry, Social Psychiatry and Psychotherapy, Hannover Medical School, Hannover, Germany; 2 Institute of Medical Psychology and Behavioral Immunobiology, University Hospital Essen, University of Duisburg-Essen, Essen, Germany; 3 Institute of Sexual Medicine and Forensic Psychiatry and Psychotherapy, Medical School, Kiel University, Kiel, Germany; 4 Forensische Genetik und Rechtsmedizin, am Institut für Hämatopathologie Hamburg GmbH, Hamburg, Germany; 5 Institute of Forensic Psychiatry, University of Duisburg-Essen, Duisburg, Germany; 6 Institute of Sexology and Sexual Medicine, Charité – Universitätsmedizin Berlin, Corporate Member of Freie Universität Berlin, Humboldt-Universität zu Berlin, and Berlin Institute of Health, Berlin, Germany; 7 Division of Mind and Brain Research, Department of Psychiatry and Psychotherapy, CCM, Charité - Universitätsmedizin Berlin, Corporate Member of Freie Universität Berlin, Humboldt-Universität zu Berlin, and Berlin Institute of Health, Berlin, Germany; 8 Division of Forensic Psychiatry, Deptartment of Psychiatry, Psychotherapy and Preventive Medicine, Ruhr University Bochum, LWL University Hospital, Bochum, Germany; 9 Department of Psychiatry, Otto-von-Guericke-University Magdeburg, Magdeburg, Germany; 10 Department of Psychiatry, University of Tübingen, Tübingen, Germany; 11 Department of Forensic Psychiatry, Ludwig Maximilians University Munich, Munich, Germany

## Abstract

Child sexual offending (CSO) places a serious burden on society and medicine and pedophilia (P) is considered a major risk factor for CSO. The androgen system is closely linked to sexual development and behavior. This study assessed markers of prenatal brain androgenization, genetic parameters of androgen receptor function, epigenetic regulation, and peripheral hormones in a 2 × 2 factorial design comprising the factors Offense (yes/no) and Pedophilia (yes/no) in analyzing blood samples from 194 subjects (57 P+CSO, 45 P−CSO, 20 CSO−P, and 72 controls) matched for age and intelligence. Subjects also received a comprehensive clinical screening. Independent of their sexual preference, child sexual offenders showed signs of elevated prenatal androgen exposure compared with non-offending pedophiles and controls. The methylation status of the androgen receptor gene was also higher in child sexual offenders, indicating lower functionality of the testosterone system, accompanied by lower peripheral testosterone levels. In addition, there was an interaction effect on methylation levels between offense status and androgen receptor functionality. Notably, markers of prenatal androgenization and the methylation status of the androgen receptor gene were correlated with the total number of sexual offenses committed. This study demonstrates alterations of the androgen system on a prenatal, epigenetic, and endocrine level. None of the major findings was specific for pedophilia, but they were for CSO. The findings support theories of testosterone-linked abnormalities in early brain development in delinquent behavior and suggest possible interactions of testosterone receptor gene methylation and plasma testosterone with environmental factors.

## Introduction

Child sexual offending (CSO) has enormous effects on children’s mental well-being and their development into adulthood^1,2^ and may affect as many as one in five children^3,4^. One major risk factor for CSO is pedophilic preference, which contributes to CSO in about 50% of convicted cases^4,5^. Neither the etiology of CSO nor that of pedophilia is well understood. Neurobiological factors have been considered in multifactorial models of both sexual preference and delinquent behavior^6^.

Androgens have been ascribed a crucial role in modulating sexual drive and function, as well as violent and aggressive behavior^7–10^. However, the relationship between androgens and behavior is complex and may depend on different external factors, as well as the age of the organism. As a result, a number of theories have been developed to explain the role of androgens under different circumstances. One of the most important and well examined is organizational–activational theory, which highlights the effects of sex steroids during critical periods of life (perinatal and puberty) leading to sustained dimorphic differentiation of the brain (organizational effects). Activational effects occur when hormones induce acute, sex-specific behaviors in adults by activating or modulating specific brain areas^11–14^. In addition, a number of other theories are the subject of discussion, such as the reciprocal model/challenge hypothesis (testosterone levels depend on specific tasks and challenges, increasing after success and decreasing after failures), the basal model (testosterone as a trait marker influencing behavior), and status theory, according to which testosterone facilitates the perception of social status and, e.g., may cause fair bargaining behavior for personal reasons (status seeking)^9,15^.

Only very crude concepts exist of when and how sexual preference might develop^4,16^. Some experts assume very early development during fetal life^16^, when organizational effects of sex steroids lead to sexual differentiation of the brain. Sexual preference might equally be categorized as sexual orientation, according to some theories^17–19^. Regarding the neurodevelopment of sexual orientation, studies have shown that androgens and an X-chromosome-linked inheritance may explain some of the variance^16,20^. In their emphasis on prenatal factors, Bao and Swaab go as far as to say, “There is no evidence that one’s postnatal social environment plays a crucial role in gender identity or sexual orientation”^16^. Clinically, pedophilia occurs almost exclusively in males, which might further support the role of male sex steroids during brain maturation. Although preliminary, small-scale studies on genetic variants in pedophiles (*n* = 13)^21^ and paraphilic sexual offenders (*n* = 97)^22^ were not able to detect alterations in a specific neurotransmitter system, they were probably underpowered to detect such a link.

Attempts to better understand the role of androgens in sexual offending have always been challenging. It has become obvious that the relationship between androgens and sexual behavior and aggression is much more complex in humans than in animal models, due to the greater impact of contextual, cognitive, emotional, and social factors. While most of the studies in this area have various methodological flaws, some have indicated a positive correlation between testosterone and/or gonadotropins and antisocial personality traits, novelty-seeking, self-reported hostility, sexual violence, sexual offence recidivism, and impulsive/aggressive behavior in child molesters and/or rapists; for a overview, see refs. ^7,8,23,24^. Another target, cysteine-adenine-guanine (CAG) repeat polymorphisms, has been investigated, with smaller numbers of CAG codon repeats being related to elevated transactivational competence of the androgen receptor protein^25,26^. As a consequence, men with shorter CAG repeats may have larger physiological effects of a certain androgen concentration than those of men with longer repeats. Studies have indicated an association of shorter CAG repeats with externalizing behaviors and male violent criminal behavior^27,28^.

All these concepts and initial empirical data show that different biological factors and the time of action need careful consideration when examining the relationship between the androgen system and sexual preference and delinquent behavior. Although we are not able to turn back the clock to explore the etiopathogenesis of sexual preference disorders and sexual offending, this cross-sectional study aimed to provide specific information on prenatal androgenization (by using the marker finger lengths ratio as a proxy), genetic parameters (CAG repeat lengths), gene×environment interactions (methylation status of the androgen receptor gene), and acute measures of the androgen system (plasma concentration of sex steroids and others). This was embedded in the careful clinical selection and characterization of subjects using a 2 × 2 factorial design with the factors pedophilia and offense status, which allowed a distinction to be made between sexual preference and offending behavior.

## Methods

### Subjects

The sample was part of the NeMUP study, as described elsewhere^29^. NeMUP is a multicenter study in Germany dedicated to disentangling the clinical, neuropsychological, and neurobiological underpinnings of pedophilia and CSO and was funded by the German Federal Ministry of Research and Education (BMBF 01KR1205). For the current analyses, 194 subjects (all Caucasian) matched for age and intelligence quotient (IQ) were included. The entire sample consisted of 57 pedophiles with child sexual offenses (P+CSO), 45 pedophiles without child sexual offenses (P−CSO), 20 non-pedophilic subjects with child sexual offenses (C+CSO), and 72 controls (C−CSO).P+CSO, P-CSO and C+CSO were collected via forum posts, email lists as well as from various outpatient departments (German Prevention Project Dunkelfel^30^) or forensic settings. All participants were comprehensively screened, as described elsewhere^29^, in order to ensure their appropriate allocation to a specific group according to sexual preference and offense status. The inclusion criterion for pedophilic men was a pedophilic and/or hebephilic preference according to the ICD-10 criteria^31^ as assessed in a a detailed sexual history together with subjective self-report data from an adapted version of the Kinsey scale for sexual fantasy and behavior^32^. Allocation to the CSO group (CSO) was based on the history of at least one sexual offence against children under the age of 14 years that involved touching or manipulating (manually or orally) the child’s naked body or genitals with the purpose of sexually stimulating himself, penetrating the child vaginally or anally, or making the child touch or manipulate the offender’s genitals or penetrate him (see for more details ref. ^29^). Exclusion criteria included intellectual disability, psychotic disorder, current severe major depressive disorder (score >15 on the Hamilton Depression Scale^33^) or anxiety disorder (score >25 in the Hamilton Anxiety Scale^34^), a clinically predominant substance misuse or dependence, and any psychotropic medication.

All participants provided written informed consent before participating. The five local ethics committees (Charite Berlin, Otto-von-Guericke-University Magdeburg, Medical School Hannover, Kiel University, Medical School, University of Duisburg-Essen) of the NeMUP research collaboration approved the study.

### Finger length ratio 2D:4D

Flat-bed scans of the left and right hand were obtained using the Canon MP Navigator EX software on a Canon LiDE210 with a resolution of 300 dpi. The distance between the tip of the finger and the ventral proximal crease of the second digit and the fourth digit was measured on black and white DIN A4 printouts by three independent raters and then averaged^35^. The intraclass correlation coefficient between the different raters was 0.716 for the right 2D:4D ratio and 0.664 for left 2D:4D ratio and 0.768 for the total 2D:4D ratio.

### Endocrine analyses

All blood samples were drawn immediately before a magnetic resonance imaging (MRI) of the brain as part of the full NeMUP investigation. Time point of blood withdrawal was not significantly different among groups, ensuring comparability and taking into account the circadian rhythms of peripheral sex steroid concentrations. About 19% of blood samples were drawn between 8 and 10 a.m., 17% between 10 and 12 a.m., 7% between 12 a.m. and 2 p.m., 26% between 2 and 4 p.m. and 20% between 4 and 6 p.m. For 11%, the exact time point was missing as no MRI was conducted. Plasma/serum concentrations of total and free testosterone, sex hormone-binding globulin (SHBG), prolactin, and cortisol were measured using enzyme-linked immunosorbent assays (IBL International, Hamburg, Germany) according to the manufacturer’s instructions. Intra-assay and interassay variances were 5.4% and 7.4%, respectively, for total testosterone; 12.3% and 8.8%, respectively, for free testosterone; 9.0 and 8.0, respectively, for SHBG; 4.6% and 5.6%, respectively, for prolactin; 3.5% and 5.0%, respectively, for cortisol. The detection limits of the assays were 0.12 ng/ml for total testosterone, 0.10 pg/ml for free testosterone, 0.77 nmol/l for SHBG, 0.35 ng/ml for prolactin, and 6.79 nmol/l for cortisol.

### DNA extraction and bisulfite conversion

Genomic DNA from whole EDTA blood was extracted by means of the QIAamp® DNA Blood Mini Kit (QIAGEN AG, Hilden, Germany), according to the manufacturer’s protocol on a Biomek Nxp (Beckman-Coulter, Krefeld, Germany). For the analysis of methylation rates, 500 ng genomic DNA from every sample was bisulfite-converted using the EpiTect® Bisulfite Kit (QIAGEN AG, Hilden, Germany). Using this procedure, unmethylated cytosines are converted into uracils via deamination (after polymerase chain reaction (PCR): thymins), whereas methylated cytosines are protected from alteration^36,37^.

### Polymerase chain reaction

#### CAGn androgen receptor polymorphism (genomic DNA)

The following primers were used for amplification of the CAGn androgen receptor polymorphism: AR-F (forward) 5′-CGCCGTCCAAGACCTACC-3′ and AR-R (reverse) 5′-GAACCATCCTCACCCTGCT-3′^38–40^. For sequencing in accordance with Sanger, 30 ng of the PCR product and AR-F were utilized in the BigDye Terminator v3.1 Cycle Sequencing Kit (Applied Biosystems, Foster City, CA, USA). The following Sequencing Standard Protocol was used for sequencing PCR: 96 °C for 1 min, 28 cycles of 96 °C for 10 s, 50 °C for 5 s, and 60 °C for 4 min. Afterwards, samples were kept at 12 °C in the instrument until further processing.

#### Methylation rates in the promotor region of the androgen receptor gene (bisulfite-treated DNA)

A fragment of 414 base pairs (from −98 to +316 bp) containing 27 CpG-sites was analyzed, covering part of a big CpG-island reaching from position −204 to +666 as defined by the “Geneious” software. Oligonucleotide primers used for the amplification PCR had the following sequences: 738_Arz_Bis_Fw: AGGGAAAAGGAGGTGGGAA (forward primer) and 784_Arz_Bis_Rev: AAATAAAAAAAAAAAAAACAAAAACAAC (reverse primer). The corresponding touchdown-PCR protocol was composed as follows: the initial taq activation step (15 min at 95 °C) was followed by 15 cycles of denaturation (30 s at 95 °C), primer annealing (40 s at 64 °C in first cycle, then 1 °C less in every following cycle), and elongation (72 °C for 40 s). Afterwards, 25 cycles followed with a denaturation temperature of 95 °C for 30 s, annealing at 54 °C for 40 s, and elongation at 72 °C for 40 s. In the end, samples were incubated at 72 °C for 4 min and kept at 12 °C until further processing or storage in the refrigerator. For sequencing PCR, again the BigDye Terminator v3.1 Cycle Sequencing Kit (Applied Biosystems, Foster City, CA, USA) was used with the forward primer and roughly 30 ng of the amplification PCR product. For sequencing PCR, the protocol “SeqATRev” was used, which entailed 96 °C for 1 min, followed by 25 cycles of 96 °C for 5 s, 60 °C for 90 s, and 50 °C for 90 s. Again, samples were kept at 12 °C in the instrument afterwards.

All sequencing was done on a Genetic Analyzer 3500 xL (Applied Biosystems).

#### Analyses of methylation rates by Epigenetic Sequencing Methylation Analysis (ESME)

Sequences were analyzed using the ESME Software (ESME). ESME determines the DNA methylation levels of sequence trace files and additionally performs quality control, normalization of signals, and correction for incomplete bisulfite conversion. Alignment of generated bisulfite and the respective reference sequences enables comparison of C to T values (in the forward sequence) and G to A values (in the reverse sequence) at CpG sites^38,41^. Exclusively CpG sites with valid results in >70% of all samples were used for the analysis.

### Statistical analyses

Statistical analysis was performed using IBM SPSS Statistics 22 (IBM, New York, NY). Results with a *p* < .05 were considered statistically significant. After testing for normal distribution, demographic, clinical, and endocrine data was analyzed using a 2 × 2 analysis of variance (ANOVA) (preference×CSO) with age and IQ as covariates. CAG repeat length data were pooled for analysis into three subgroups, resulting in subjects with short (7–18), medium (19–23), or long (24–32) CAG repeat length^38,42^. For methylation analysis, CpG positions with a variance of <0.001 over the whole sample were excluded from further analysis. Through this procedure, two CpG positions were discarded (−054 and +068). We then performed a linear mixed models analysis using a restricted estimated maximum likelihood approach with two hierarchical levels subjects and the repeated measure within-subject factor CpG site over the remaining 25 CpG positions. DNA methylation was computed as the dependent variable and CpG site, CAG repeats, pedophilia, and offense status as fixed factors. Interactions between CpG site and other factors, as well as between CAG repeats and other factors in the model, were also assessed. The relation between methylation and hormones was analyzed by correlating mean methylation over the whole analyzed sequence and concentrations of hormones.

## Results

### Clinical characteristics of the study groups

The four groups did not differ in age (range 36.8–40.5 years) or intelligence (estimated IQ between 93.8 and 103.2). Clinical examination revealed significantly higher levels of impulsivity as assessed by the Barret Impulsivity Scale (BIS−CSO: 62.0 ± 7.9, BIS+CSO: 65.0 ± 10.9) in child sexual offenders (main effect of offense status; *F*(1,182) = 5.537, *p* = .02, *η*^2^ = 0.03), and elevated levels of own perceived traumatization and neglect during childhood as assessed by the Childhood Trauma Questionnaire (main effect of offense status; *F*(1,184) = 29.88, *p* < .001, *η*^2^ = 0.14; interaction effect between offender and pedophilia (*F*(1,184) = 7.33, *p* = .007, *η*^2^ = 0.038; Table 1).

**Table 1:** Raw data (mean ± standard deviation) of the clinical characterization for the different subgroups investigated, including statistical *p* values of a 2 × 2 ANOVA with the factors offense status (+CSO/−CSO) and pedophilia (+P/−P)

Parameter/group	P+CSO	P−CSO	−P+CSO	−P−CSO	*p* Value (CSO/P/CSO×P)
Age (*n* = 194)	39.5 ± 8.7	36.8 ± 9,1	40.5 ± 11.8	38.2 ± 9.9	.095/.495/.971
IQ (*n* = 193)	99.8 ± 16.3	103.2 ± 14.27	93.8 ± 12.72	99.96 ± 18.56	.072/.082/.607
Smokers (S/N/MD)	18/11/28	10/14/21	11/3/6	18/24/30	.011*/.913/—
BIS (*n* = 186)	64.14 ± 10.74	63.54 ± 8.13	67.47 ± 11.13	61.06 ± 7.73	.02*/.776/.052
CTQ (*n* = 188)	50.3 ± 19.7	43.3 ± 14.3	57.0 ± 16.7	36.4 ± 11.9	<.001*/.972/.007*
Violent crimes (mean ± std, *n* = 184)	0.18 ± 0.468	0.09 ± 0.288	0.4 ± 1.142	0.08 ± 0.441	.019*/.262/.196
Sexual crimes (mean ± std, *n* = 184)	3.51 ± 3.671	0.07 ± 0.33	2.1 ± 3.177	0 ± 0	<.001*/.046*/.069
Other crimes (mean ± std, *n* = 184)	0.62 ± 1.229	0.5 ± 1.577	1.6 ± 2.415	0.77 ± 4.92	.355/.246/.52

*p* Values are depicted in the following order: (main effect of offense/main effect of pedophilia/interaction effect of offense×pedophilia)

*S* smokers, *N* non-smokers, *MD* missing data

* denotes significant results of the ANOVA with p < 0.05

### Endocrine parameters

**Fig. 1:**
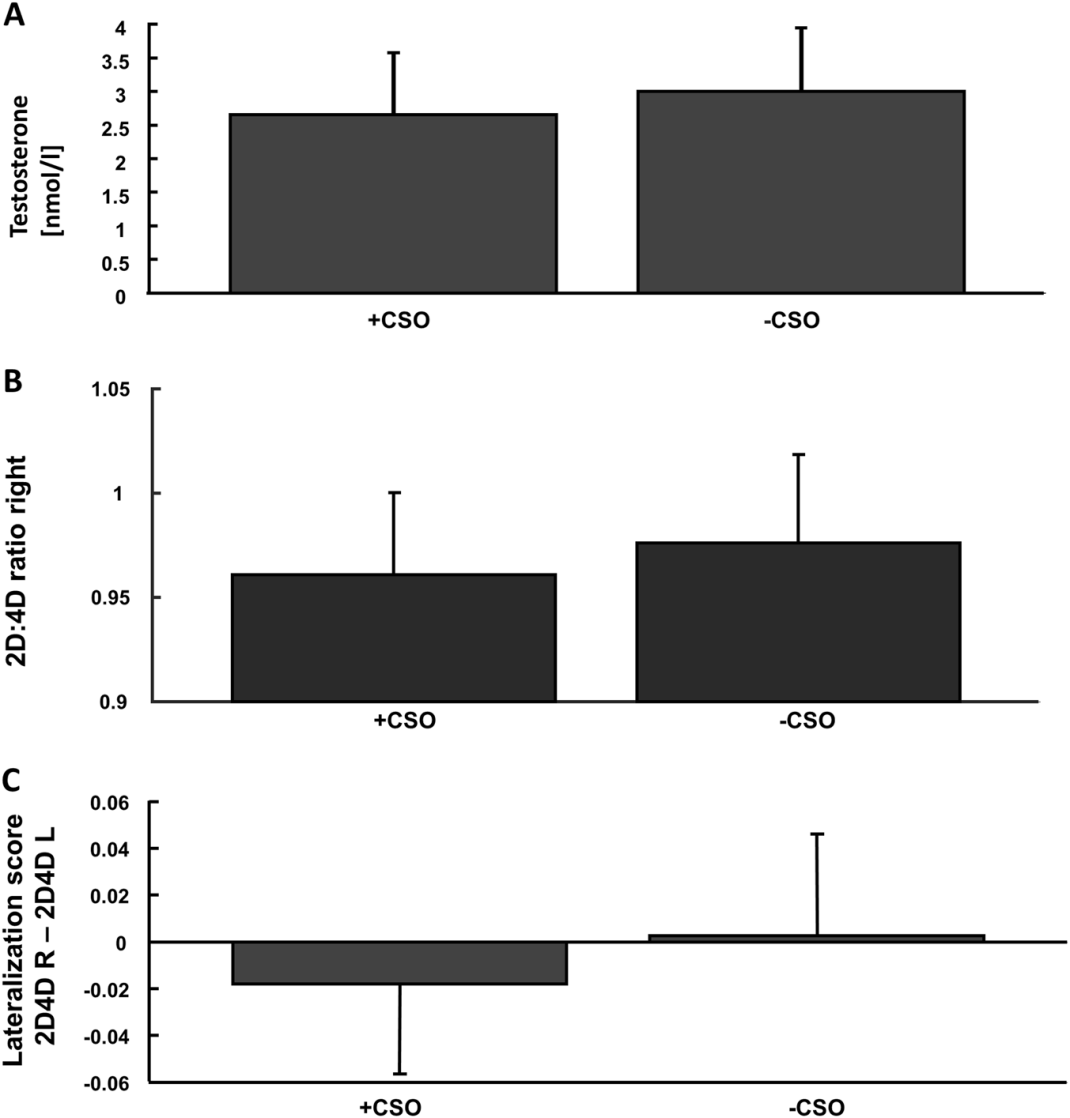
Testosteron levels & markers of prenatal andorgenization. **a** Acute levels of peripheral testosterone (mean ± std): child sexual offenders (+CSO) showed significantly lower levels compared with non-child sexual offenders (−CSO), as indicated by a 2 × 2 ANOVA with the factors pedophilia and CSO. **b** Digit-to-ring finger ratio (2D:4D) of the right hand: child sexual offenders (CSO) had a lower 2D:4D ratio of the right hand compared with non-child sexual offenders (−CSO), indicating a higher level of prenatal testosterone. **c** Lateralization score of the right hand: child sexual offenders showed significantly lower lateralization scores as an additional measure of prenatal androgenization

In order to examine the different hypotheses on the role of sex and corticosteroid hormones in regulating behavior as outlined above, different endocrine parameters were analyzed. These analyses revealed lower testosterone levels in child sexual offenders for total testosterone (main effect of offense status: *F*(1,172) = 4.84, *p* = .029, *η*^2^ = 0.027) and borderline effects for free testosterone (main effect of offense status: *F*(1,172) = 2.96, *p* = .087, *η*^2^ = 0.017; Table 1 and Fig. 1a). For prolactin levels, we detected an interaction effect between offense status and sexual preference (*F*(1,172) = 8.04, *p* = .005, *η*^2^ = 0.045). Finally, there was a marginally lower testosterone/cortisol ratio in child sexual offenders compared with non-offending subjects (*F*(1,172) = 3.39, *p* = .067, *η*^2^ = 0.019; Table 2).

**Table 2:** Raw data (mean ± standard deviation) of the endocrine parameters for the different subgroups investigated, including statistical *p* values of a 2 × 2 ANOVA with the factors offense status (+CSO/−CSO) and pedophilia (+P/−P)

Parameter/group	P+CSO	P−CSO	−P+CSO	−P−CSO	*p* Value (CSO/P/CSO×P)
Testosterone (*n* = 176)	2.62 ± 0.97	3.13 ± 0.86	2.74 ± 0.81	2.91 ± 0.99	.029*/.739/.289
Free testosterone (*n* = 176)	4.25 ± 2.87	5.02 ± 2.15	3.79 ± 1.69	4.95 ± 4.53	.087/.64/.737
Prolactin (*n* = 176)	9.38 ± 4.97	8.58 ± 4.87	6.22 ± 2.38	9.58 ± 4.02	.083/.145/.005*
Cortisol (*n* = 176)	683.61 ± 271.58	689.97 ± 271.06	658.74 ± 222.03	700.7 ± 310.55	.88/.705/.705
Testosterone/cortisol ratio	0.0042 ± 0.0017	0.0052 ± 0.0023	0.0046 ± 0.0023	0.0050 ± 0.0029	.067/.747/.436

*p* Values are depicted in the following order: (main effect of offense/main effect of pedophilia/interaction effect of offense×pedophilia)

* denotes significant results of the ANOVA with p < 0.05

### Indicators of prenatal androgenization

2D:4D ratios were analyzed to test whether a higher prenatal testosterone level is connected to child sexual offending or pedophilia. Proxy markers of prenatal androgen exposure indicated a directional asymmetry (right–left difference) of 2D:4D digit ratio in child sexual offenders (*F*(1,166) = 6.41, *p* = .012, *η*^2^ = 0.037), as well as a trend toward smaller 2D:4D ratios of the right hand (*F*(1,166) = 3.7, *p* = .056, *η*^2^ = 0.022). For left hand 2D:4D ratios and mean 2D:4D ratios (mean of both hands), interaction effects were found (left: *F*(1,166) = 5.53, *p* = .02, *η*^2^ = 0.032; mean: *F*(1,166) = 4.35, *p* = .039, *η*^2^ = 0.026) (Table 3, and Fig. 1b, c).

**Table 3:** Raw data (mean ± standard deviation) of prenatal androgenization (2D:4D ratio) for the different subgroups investigated, including statistical *p* values of a 2 × 2 ANOVA with the factors offense status (+CSO/−CSO) and pedophilia (+P/−P)

Parameter/group	P+CSO	P−CSO	−P+CSO	−P−CSO	*p* Value (CSO/P/CSO×P)
2D:4D ratio right (*n* = 170)	0.961 ± 0.040	0.966 ± 0.042	0.960 ± 0.040	0.982 ± 0.042	.056/.271/.217
2D:4D ratio left (*n* = 170)	0.983 ± 0.040	0.964 ± 0.032	0.969 ± 0.038	0.979 ± 0.039	.479/.873/.020*
2D:4D ratio mean (*n* = 170)	0.972 ± 0.034	0.965 ± 0.03	0.965 ± 0.036	0.981 ± 0.034	.434/.442/.039*
2D:4D (R–L) (*n* = 170)	-0.022 ± 0.042	0.002 ± 0.045	-0.009 ± 0.027	0.003 ± 0.043	.012*/.347/.371

*p* Values are depicted in the following order: (main effect of offense/main effect of pedophilia/interaction effect of offense×pedophilia)

* denotes significant results of the ANOVA with p < 0.05

### Epigenetic and genetic analyses

**Fig. 2:**
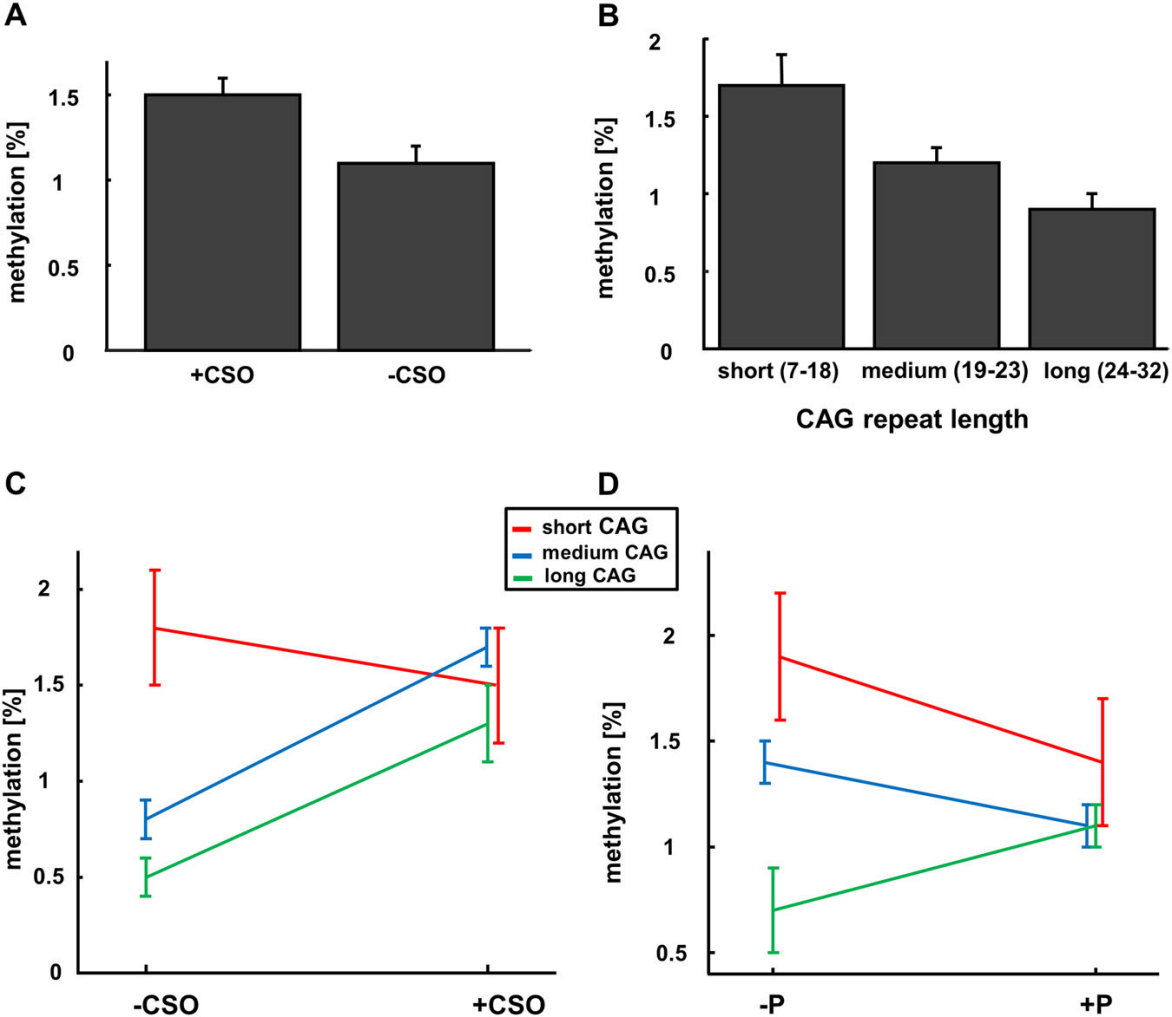
Methylation levels of the androgen receptor (mean ± std) **a** Mixed model analysis showed higher methylation levels in child sexual offenders (+CSO) compared with non-child sexual offenders in general (−CSO), as well as **b** lower methylation levels of the androgen receptor in subjects with long CAG repeat length and **c** interaction between offending status and CAG repeat length. In the non-offending population, carriers of short CAG repeat length showed the highest methylation levels, while carriers with medium and long CAG repeat length showed low levels of methylation. In the offending group, however, no differences in methylation levels between different CAG repeat lengths were found. **d** Interaction between pedophilia and CAG repeat length on methylation levels. For the non-pedophilic subgroup, lowest methylation levels were found for carriers with long CAG repeat length. For the pedophilic subgroup, no such differences could be found for different CAG repeat length

As in previous studies, we use a linear mixed model analysis to explore the relationship between epigenetic and genetic markers with offense status and sexual preference. These analyses revealed main effects of offense status (*F*(1,4633) = 6.02, *p* = .014), CpG position (*F*(24,4633) = 4.28, *p* < .001), and CAG repeat length (*F*(2,4633) = 6.94, *p* = .001) on methylation levels (Fig. 2a, b). In addition, there were interaction effects between CAG repeat length and offense status (*F*(2,4633) = 3.48, *p* = .031; Fig. 2c) and between CAG length and pedophilia (*F*(2,4633) = 4.08, *p* = .017; Fig. 2d) on methylation levels.

**Fig. 3:**
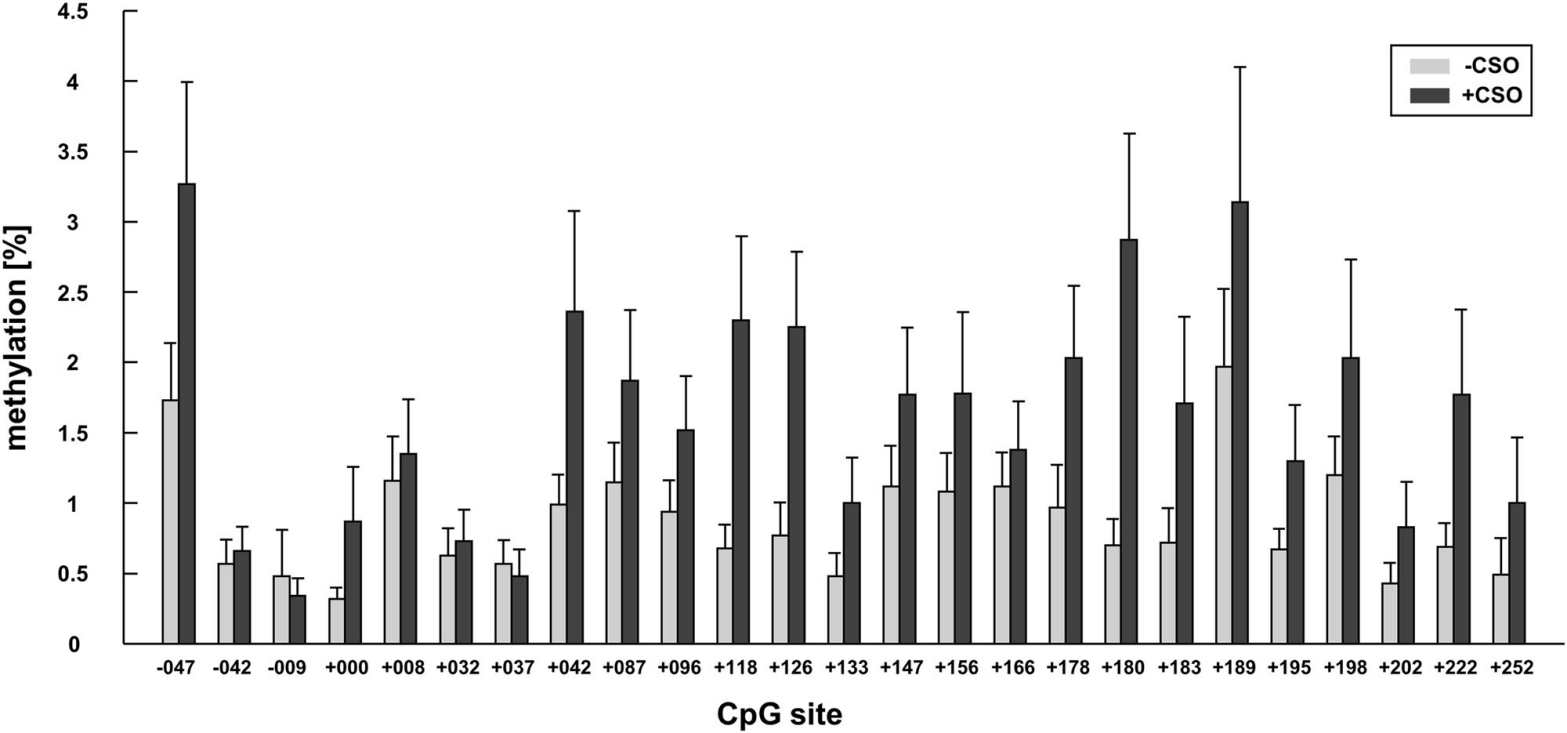
Methylation levels for the single CpG sites are shown for offenders and non-offenders (mean ± std)

Bonferroni corrected post hoc *t* test showed that offending subjects had higher methylation levels than those of non-offending subjects (CSO: 0.015 ± 0.001; −CSO: 0.011 ± 0.001, *p* = .014; Figs. 2a and 3). For the CAG repeat length, Bonferroni corrected *t* test showed that subjects with long CAG repeat length had lower methylation levels than those of subjects with short and medium CAG repeat length (long: 0.009 ± 0.01; medium: 0.012 ± 0.001, *p* = .04; short: 0.017 ± 0.002, *p* = .001; Fig. 2b and Table 4), while the difference between short and medium showed only a trend toward significance (*p* = .083).

**Table 4:** Raw data (mean ± standard deviation) of CAG repeat length (2D:4D ratio) for the different subgroups investigated, including statistical *p* values of a 2 × 2 ANOVA with the factors offense status (+CSO/−CSO) and pedophilia (+P/−P)

Parameter/group	P+CSO	P−CSO	−P+CSO	−P−CSO	*p* Value (CSO/P/CSO×P)
CAG repeats	21.5 ± 3.7	22.5 ± 2.6	22.2 ± 2.6	22.2 ± 3.4	.343/.739/.390
CAG length (short/medium/long)	7/32/17	2/24/16	1/14/5	6/41/24	.526/.879/—

*p* Values are depicted in the following order: (main effect of offense/main effect of pedophilia/interaction effect of offense×pedophilia)

In addition, a negative correlation between CAG repeat length and methylation levels was found only in the non-offending group (*r* = −.204, *p* = .03), while neither the whole sample (*r* = −.074, *p* = .316) nor the offending group (*r* = .069, *p* = .553) showed this correlation.

### Correlational analyses between biological and behavioral parameters

To evaluate the behavioral aspects associated with methylation of the androgen receptor, correlations between methylation and clinical characteristics, hormone levels, and prenatal androgenization were accessed. Correlational analyses revealed a significant inverse association between the total number of sexual offenses and 2D:4D ratios of the right hand (*r* = −.184, *p* = .018), left hand (*r* = −.177, *p* = .022), and mean of left and right hand (*r* = −.213, *p* = .006). Moreover, the total number of sexual offenses was positively correlated with mean methylation of the androgen receptor gene (*r* = .193, *p* = .008).

## Discussion

**Fig. 4:**
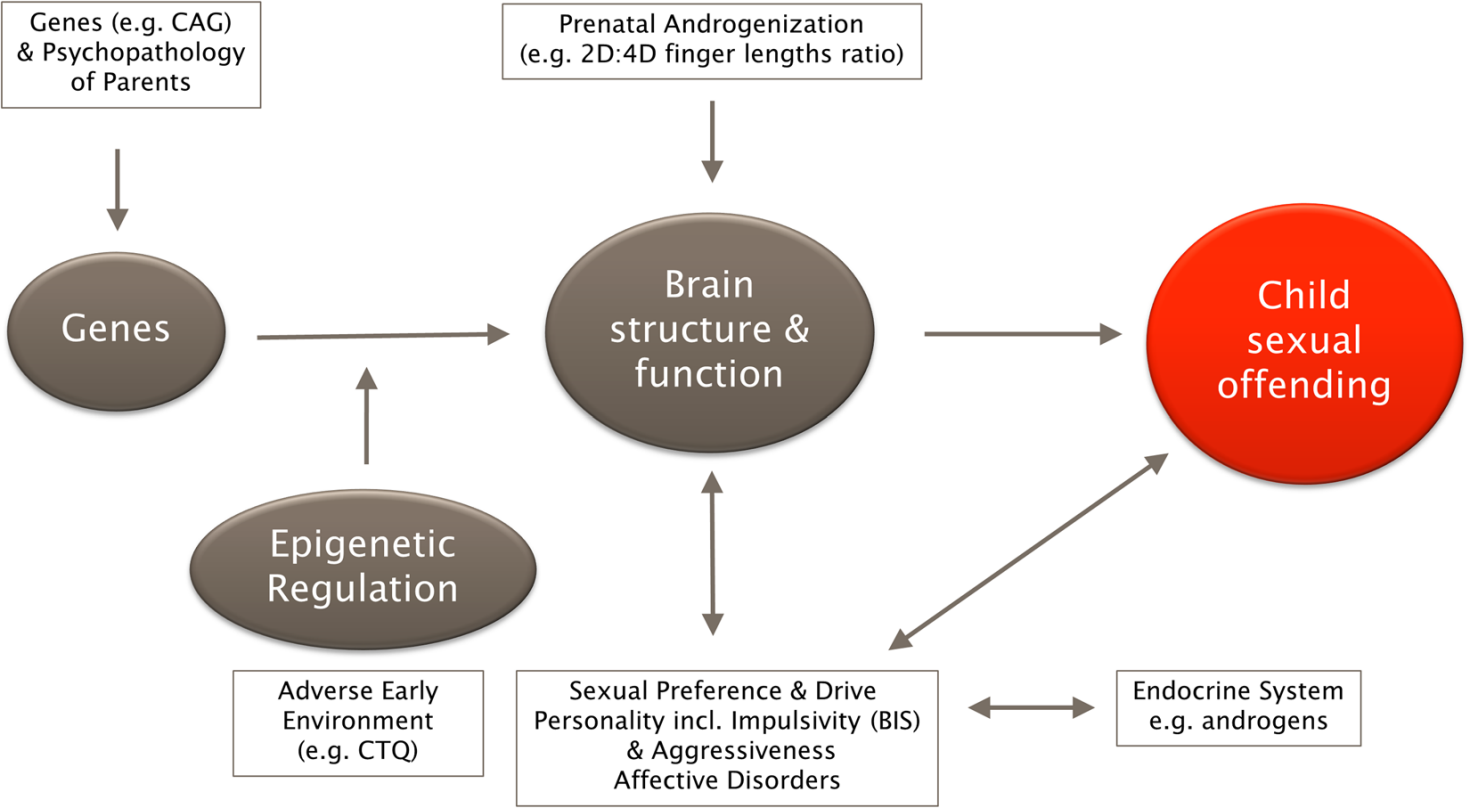
Neurobiological working model of child sexual offending. We assume an underlying combination of genetic, epigenetic, and environmental factors that contribute to child sexual offending. A high level of prenatal testosterone or childhood trauma may lead to a disruption of the regulatory circuit between genetics and epigenetics. This in turn could lead to difficulties for the subject in adapting adequately to environmental factors, which then could foster the development of personality or affective disorder, as well as impulsive behavior

This is the first and largest study providing a detailed investigation of prenatal, genetic, and epigenetic parameters of the androgen system in a comprehensively examined sample of child sexual offenders with and without pedophilia, non-offending pedophiles, and controls. While pedophilia per se was not associated with sustained abnormalities of the androgen system, CSO was related to a number of behavioral, prenatal, and epigenetic alterations. We propose a working model that can serve as a basis for discussion of these results, as well as the further refinement and development of theories of neurobiological and environmental factors, on the road to CSO (see Fig. 4).

With regard to previous empirical data and models, the current data set fits the conception of a prenatally more masculinized brain (assessed here by a proxy marker) in CSO, prone to externalizing behaviors and disorders^43–46^, as well as forms of direct and indirect aggression^47–49^ and dominance^50^. Main effects of offense status were seen for directional asymmetry and marginally for right hand ratios, whereas mean and left hand ratios showed interaction effects that might also indicate a role in the development of sexual preference. Differences between right and left hand ratios have been discussed elsewhere, with some evidence pointing toward stronger correlates of prenatal testosterone for right 2D:4D and right–left 2D:4D (directional asymmetry) than for left hand ratios^51–53^. Intriguingly, these proxy markers were correlated with total number of child sexual offenses, which may further support the role of prenatal androgenization in the development of delinquent behaviors.

In terms of genetic factors, there were no main effects of androgen receptor polymorphism (CAG repeat lengths) on either offense status or sexual preference at a group-level analysis (ANOVA). However, mixed models analyses indicated significant effects of CAG repeat lengths for the extent of androgen receptor gene methylation. On the one hand, shorter CAG repeats were associated with a higher percentage of methylation (Fig. 2b). On the other, offending subjects generally displayed higher levels of methylation (see Fig. 2a), while a small group of non-offending subjects with short CAG repeat length had higher levels of methylation than those with medium or long CAG repeat length carriers (Fig. 2c). In non-offending subjects, androgen receptor protein synthesis (as indicated by methylation level) was negatively correlated with androgen receptor functionality (as indicated by CAG repeat length), indicating a regulatory mechanism (i.e., the higher the receptor functionality, the less androgen receptors are synthesized) for maintaining a normal functioning system. Interestingly, in the offending population these relations were not apparent, suggesting a compromised regulatory mechanism between genetic and epigenetic factors in the androgen system.

Finally, correlational analyses revealed a negative association between markers of prenatal androgenization and number of child sexual offenses, as well as a positive association between methylation level and total number of child sexual offenses. Thus one might speculate that an elevated prenatal androgenization of the brain compromises the regulatory abilities of the androgen system to adapt to environmental factors in later life.

Although some of these results warrant replication because of the small sample size of the subgroups, it might be speculated that higher levels of methylation are an attempt by the organism to downregulate a highly functional androgen (receptor) system and/or downregulate androgen-driven impulsivity and aggressive behaviors toward children in those subjects who have committed CSO in the past but are currently willing to control their behaviors. As mentioned below, our samples consisted predominantly of men recruited from outpatient departments and forensic settings, most of whom were motivated not to reoffend. In line with our interpretation, peripheral concentrations of plasma testosterone and testosterone/cortisol ratios were lower in offending than in non-offending subjects, which may reflect a restrictive and well-controlled coping strategy.

Comparative studies exploring the interaction between organizational and activational effects of the androgen system are scarce or even non-existent. Previous studies of normal men have failed to find an influence of the androgen receptor gene on right hand 2D:4D ratio, with men with more sensitive androgen receptors even tending to score lower on subscales of aggression questionnaires^54,55^.

### A neurobiological working model of child sex offending

There are a number of studies that have investigated the neurobiological underpinnings of pedophilic disorders; for a critical review, see refs.^56,57^. However, most of them failed to distinguish between sexual preference and offense status, making it difficult to draw conclusions regarding the origins of pedophilia and/or offending behavior. Only recently, the NeMUP consortium has taken into account the differential effects of these two factors. This has produced a series of results indicating more normal than abnormal findings regarding the neuropsychological and neurobiological factors underlying pedophilia. Most importantly, the majority of findings were attributable to CSO and included worsened response-inhibition abilities^58^ and lower right temporal pole volumes in *offending pedophiles*^59^, as well as superior inhibitory control (Go/No-Go paradigm) accompanied by inhibition-related activation in the left posterior cingulate and left superior frontal cortex in *non-offending pedophiles*^60^. These findings, together with our current findings on the androgen system, can be implemented in a working model of neurobiological factors contributing to CSO (Fig. 4). While there is no evidence of a monocausal etiology of pedophilia and/or CSO, the model highlights multiple components that may exceed a threshold above which offending behavior becomes increasingly possible. While none of these contributory factors by itself is specific to CSO, several of them might increase the probability of its occurrence, pedophilic preference being one of the most important. This working model may be helpful for the further development of research questions and more elaborate analyses of existing and forthcoming data sets, as well as improvements and adaptations of prevention and treatment programs.

### Limitations and suggestions for future studies

1. Although NeMUP provides one of the largest and best-examined samples of men with and without pedophilia and CSO, the CSO−P group was relatively small because access to and recruitment of these subjects is extremely challenging. Nevertheless, by using a two-by-two factorial design the group of child sexual offenders (irrespective of sexual preference) was sufficiently large to draw conclusions.
2. Careful clinical and psychometric examination of these subjects^29^ was carried out which revealed higher levels of impulsivity and experienced neglect and traumatization in child sexual offenders compared to non-child offenders. However, apart from the number of offenses, which actually showed a very intriguing association with prenatal and epigenetic parameters, other measures showed no significant associations. Additional parameters on, e.g., direct and indirect aggression, as well as other static and dynamic risk factors, might have led to additional results that were not apparent in this data set.
3. The assessments carried out here provide only a snapshot of the role of the androgen system and thus may not entirely reflect what has happened in the past or is currently happening in these subjects. Proxy markers such as 2D:4D finger length ratios may be helpful, but they can be affected by many factors and diseases and therefore may be rather non-specific or of questionable reliability (for meta-analysis, see ref. ^49^). Additionally, data on metabolites of testosterone in the brain, such as estradiol and dihydrotestosterone, and their respective enzymes aromatase and 5α-reductase, were not assessed and could be of value in future analyses.
4. Measurement of peripheral hormones for analyzing activational effects may have only limited validity in a cross-sectional study with no specific experimental intervention, since many factors may acutely influence endocrine parameters (e.g., stress, sleep quality, fatigue, hunger, and time of blood withdrawal).
5. Many participants were drawn from outpatient departments or forensic settings, which might have led to a bias in our sample toward people who seek help and show cooperation, thereby directly affecting epigenetic and endocrine processes.
6. Taken together, these initial results may point toward the importance of the androgen system for a better understanding of the different pathways toward sexual violence. Prenatal and epigenetic measures combined with a careful clinical characterization appear promosing tools for future studies, which may also focus on different forms of sexual violence, e.g., against children vs. against adults.
