# Author Correction: Child sexual offenders show prenatal and epigenetic alterations of the androgen system

**DOI:** 10.1038/s41398-019-0417-6

**Published:** 2019-02-18

**Authors:** Tillmann H. C. Kruger, Christopher Sinke, Jonas Kneer, Gilian Tenbergen, Abdul Qayyum Khan, Alexandra Burkert, Linda Müller-Engling, Harald Engler, Hannah Gerwinn, Nicole von Wurmb-Schwark, Alexander Pohl, Simone Weiß, Till Amelung, Sebastian Mohnke, Claudia Massau, Christian Kärgel, Martin Walter, Kolja Schiltz, Klaus M. Beier, Jorge Ponseti, Boris Schiffer, Henrik Walter, Kirsten Jahn, Helge Frieling

**Affiliations:** 1 Department of Psychiatry, Social Psychiatry and Psychotherapy, Hannover Medical School, Hannover, Germany; 2 Institute of Medical Psychology and Behavioral Immunobiology, University Hospital Essen, University of Duisburg-Essen, Essen, Germany; 3 Institute of Sexual Medicine and Forensic Psychiatry and Psychotherapy, Medical School, Kiel University, Kiel, Germany; 4 Forensische Genetik und Rechtsmedizin, am Institut für Hämatopathologie Hamburg GmbH, Hamburg, Germany; 5 Institute of Forensic Psychiatry, University of Duisburg-Essen, Duisburg, Germany; 6 Institute of Sexology and Sexual Medicine, Charité-Universitätsmedizin Berlin, Corporate Member of Freie Universität Berlin, Humboldt-Universität zu Berlin, and Berlin Institute of Health, Berlin, Germany; 7 Division of Mind and Brain Research, Department of Psychiatry and Psychotherapy, CCM, Charité-Universitätsmedizin Berlin, Corporate Member of Freie Universität Berlin, Humboldt-Universität zu Berlin, and Berlin Institute of Health, Berlin, Germany; 8 Division of Forensic Psychiatry, Deptartment of Psychiatry, Psychotherapy and Preventive Medicine, Ruhr University Bochum, LWL University Hospital, Bochum, Germany; 9 Department of Psychiatry, Otto-von-Guericke-University Magdeburg, Magdeburg, Germany; 10 Department of Psychiatry, University of Tübingen, Tübingen, Germany; 11 Department of Forensic Psychiatry, Ludwig Maximilians University Munich, Munich, Germany

**Correction to: Translational Psychiatry;**

10.1038/s41398-018-0326-0;Published online 18 Jan 2019.

The affiliations. Originally, Kolja Schilz was named last in the affiliations, implying that he is the senior author. This has been corrected; Kolja Schilz is now mentioned after Martin Walter in both the html and PDF versions of the article.

